# Structural prediction of host-guest structure in lithium at high pressure

**DOI:** 10.1038/s41598-018-23473-5

**Published:** 2018-03-27

**Authors:** Prutthipong Tsuppayakorn-aek, Wei Luo, Teeraphat Watcharatharapong, Rajeev Ahuja, Thiti Bovornratanaraks

**Affiliations:** 1 Extreme Conditions Physics Research Laboratory (ECPRL) and Physics of Energy Materials Research Unit (PEMRU), Department of Physics, Faculty of Science, Chulalongkorn University, 10330 Bangkok, Thailand; 2 Thailand Center of Excellence in Physics, Commission on Higher Education, 328 Si Ayutthaya Road, Bangkok, 10400 Thailand; 3 Condensed Matter Theory Group, Department of Physics and Astronomy, Uppsala University, Box 516, 75120 Uppsala, Sweden; 4 Department of Materials and Engineering, Applied Materials Physics, Royal Institute of Technology (KTH), SE-100 44 Stockholm, Sweden

## Abstract

*Ab initio* random structure searching (AIRSS) technique is used to identify the high-pressure phases of lithium (Li). We proposed the transition mechanism from the fcc to host-guest (HG) structures at finite temperature and high pressure. This complex structural phase transformation has been calculated using *ab initio* lattice dynamics with finite displacement method which confirms the dynamical harmonic stabilization of the HG structure. The electron distribution between the host-host atoms has also been investigated by electron localization function (ELF). The strongly localized electron of p bond has led to the stability of the HG structure. This remarkable result put the HG structure to be a common high-pressure structure among alkali metals.

## Introduction

Lithium (Li) is one of the most fascinating element in the periodic table and continues to attract a lot of attention due to the outstanding properties suitable for several applications, for example, battery technology^1^, hydrogen storage^2^, high electrical resistance^3^, high superconducting transition temperature^4^. Moreover, Li is one of central interest in many disciplines, especially in high pressure physics. It is the huge interest in the theoretical study^5^ and the experimental observation^6^ as there is the stability of some of the complex structure. While the high-pressure phase of alkali metals, namely, sodium, potassium, and rubidium, have also been found to be the complex structure. The complex structure of alkali metals were reported to have the host-guest (HG) structure^7–9^. Interestingly, one important question that remains unresolved is the HG structure in Li at high pressure. Recently, T. Matsuoka *et al*^10^. reported structural phase transitions in lithium (Li) using experimental apparatus. The reported transition sequence is *Im*$\bar{3}$*m* (0–8 GPa) → *Fm*$\bar{3}$*m* (8–39 GPa) → *R*$\bar{3}$*m* (39–44 GPa) → *I*$\bar{4}$3*d* (44–73 GPa) → *C*2*mb* (73–80 GPa) → *C*2*cb* (80–120 GPa) → *Cmca* (>120 GPa)^6,11–13^. However, it does not have the HG structure at any pressure among such sequence.

Goncharov *et al*.^14^ have suggested that Li can be an incommensurate HG structure since crystal structure at the pressure above 50 GPa has never been unveiled. They suggested the distances between metal atoms in the incommensurate guest chains may be substantially shorter than expected, causing an increase of the phonon frequency because the host modulates the guest atom positions. However, a clear explanation based on the theoretical aspect has not yet been achieved. Among structural prediction in the crystal structures of materials, Li is one of the most from interested structural predictions at high pressure^5,15–17^. The theoretical study revealed novel structures in Li^15–17^ at high pressure but they have not found the HG structure. Hence, the question regarding the stability of HG structure can be related to the broader issue, namely, the importance of the accuracy of *ab initio* calculation used in the prediction.

In a recent theoretical study by Pickard and Needs^18^, the low-pressure close-packed structures were reported to be able to transform into the HG structure at terapascal pressures. They have shown that the formation of *s*-*p* hybridized bonds described by a two-component model consisting of positive ions and interstitial electron *blobs* at very high pressures. Interest in the formation of *s*-*p* hybridized bonds began to point out that it can be an incommensurate HG structure^14^. At this point, the observed accumulations of electrons on the interstitial sites can determine the stable structure.

In this study, we explore an existence of HG in Li metal by performing *ab initio* random structure searching (AIRSS) method^19^. This approach is predicted the structure which is confirmed the previously proposed experimental observation^14^. Moreover, we present that the discoveries of the transitions sequence can lead to the new interpretation of Li. We report the stable structure through the observation of the formation of *s*-*p* hybridized bonds in Li. The importance of this result is that it clearly shows the formation mechanisms of the HG structure at high pressure. Our findings provide a significant advance in the understanding of the behavior of solid Li at high pressure.

## Results and Discussion

**Figure 1:**
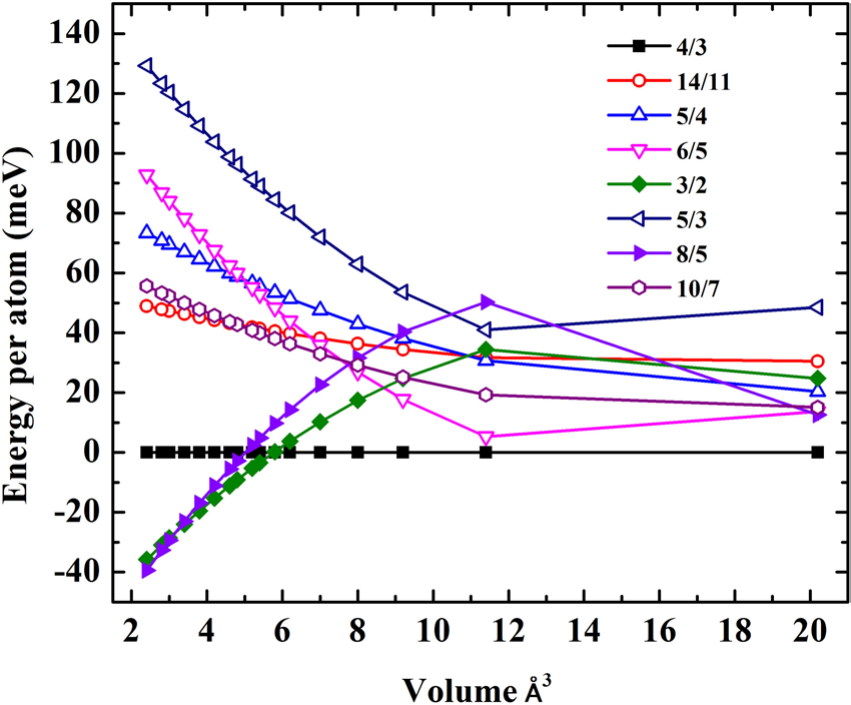
Total energies of various commensurate analogues with respect to the energy of the commensurate supercell with commensurate value *c*_*H*_/*c*_*G*_ = 4/3 as a function of volume/atom.

We find the experimentally observed unknown phase to be stable in a range of pressure above 50 GPa^14^. Based on structural predictions using AIRSS technique, we have obtained the lowest-enthalpy crystal structure with space group *I*4/*mcm*. We refer the predicted stable structure that the *I*4/*mcm* is the periodic approximations to the incommensurate host-guest structure as the new structure of high-pressure Li of *I*4/*mcm* symmetry is composed of two structures. In fact, it is well known that each of the host-guest structures has the same host framework consisting of eight-atom units, and the number of guest atoms varies. The HG structure is described by approximating with a commensurate supercell along the *c* axis. Thus, based on this structural model, the definition of approximating with a commensurate supercell along the *c* axis can be written as *c*_*H*_/*c*_*G*_ where c_*H*_ is the host lattice along the *c* axis and *c*_*G*_ is the guest lattice along the *c* axis. To treat the parameter of *c*_*H*_/*c*_*G*_, the HG structure has kept the value of *c*_*H*_/*c*_*G*_ channels in along the *c* axis of the host structure. The lowest total energy of the HG structure is calculated by approximating with a commensurate supercell along the *c* axis. This is due to the fact that the HG structure is the aperiodic structure along the *c* axis of the host structure as the component of the guest structure made up of chains that lie in this channel. Therefore, it is well known that the guest structure is an incommensurate with the host structure. In order to accurately calculate the dependence of *c*_*H*_/*c*_*G*_ of the HG on applied pressure, we present the stability of the HG structure through various commensurate analogues with respect to the *c*_*H*_/*c*_*G*_ = 4/3. By approximating the complex HG structure, *c*_*H*_/*c*_*G*_ = 4/3, a commensurate supercell of the host structure is generated 3c_*H*_ and also the guest structure with 4c_*G*_. We choose the following sets of the parameter for *c*_*H*_/*c*_*G*_: 4/3, 14/11, 5/4, 6/5, 3/2, 5/3, 8/5, and 10/7, which yield two kinds of structures as far as symmetry is involved. Our calculations determine a stable structure by calculating total energies of various commensurate analogues with respect to the energy of *c*_*H*_/*c*_*G*_ = 4/3 commensurate supercell as a function of volume per atom, which can be seen in Fig. 1. Our calculations indicate a dramatic increase in the energy difference of the HG structure. The *c*_*H*_/*c*_*G*_ = 4/3 is the most energetically stable over a wide range of volumes. At the maximum pressure, it shows the compressibility transformation of the HG structure. With increasing pressure, our calculation propose an incommensurate-to-incommensurate phase transition by examining the *c*_*H*_/*c*_*G*_. In the case of the HG structure, we have predicted that the 4/3 (1.333) transforms into the 3/2 (1.5) and transforms into the 8/5 (1.6), respectively.

**Figure 2:**
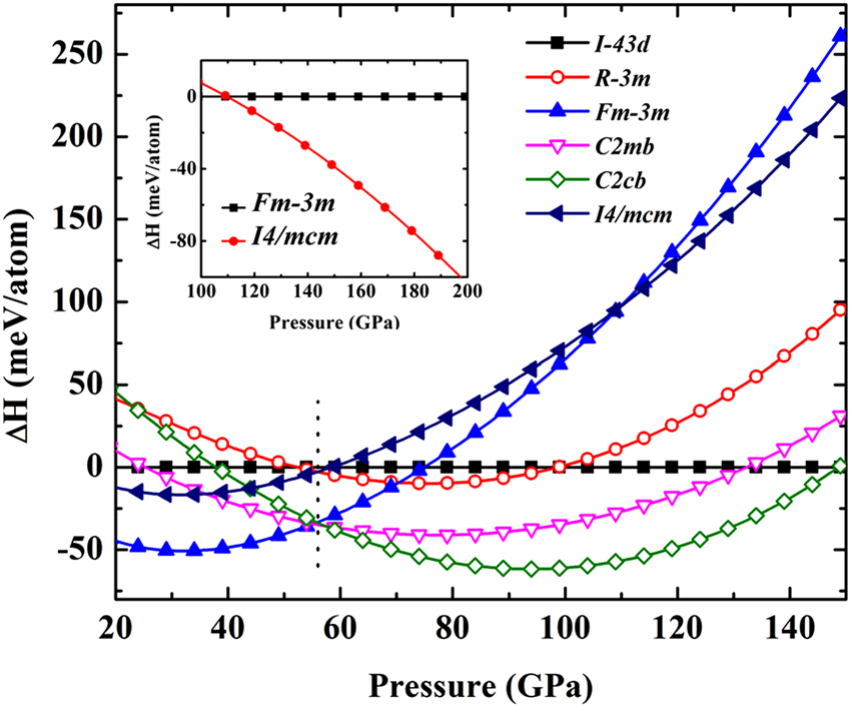
Comparison of the enthalpies of Li phases up to 150 GPa. Li structures relative to the *I*$\bar{4}$3*d* structure at 0 K. The inset shows the enthalpy of the fcc structure with the *I*4/*mcm*.

From full details of the total energies of various commensurate procedures, we use the commensurate value of 4/3 for describing the stable structure. Enthalpies of different phases are shown with respect to the enthalpy of the the *I*$\bar{4}$3*d* as can be seen in Fig. 2. We have precisely indicated that it is the thermodynamically stable structure over a wide range of pressures. We find that the crystal structure of Li exhibits different origins in two-phase transition sequences at the pressure around 50 GPa as shown below:

1 $${Simple}:{Fm}-3{m}\,\mathop{\longrightarrow }\limits^{55{GPa}}\,{C}2{mb}\,\mathop{\longrightarrow }\limits^{56{GPa}}\,{C}2{cb}$$

2 $${Complex}:{I}-43{d}\,\mathop{\longrightarrow }\limits^{52{GPa}}\,{R}-3{m}\,\mathop{\longrightarrow }\limits^{56{GPa}}\,I4/{mcm}$$

The first transition sequence is associated with the simple structural phase transformation in which the fcc structure transforms into the *C*2*mb* structure and then the *C*2*cb* structure, respectively. While the *C*2*mb* is stable in a very narrow pressure range, the *C*2*cb* structure is revealed to be the most enthalpy favourable. The second transition sequence is relevant to the complex structural phase transformation at finite temperature by determining enthalpy-pressure (Fig. 2), in which the *I*$\bar{4}$3*d* structure transforms into the *R*$\bar{3}$*m* structure and then the *I*4/*mcm* analogue. This result opens up the possibility that different origins in two-phase transition sequences may allow the *I*4/*mcm* structure to be energetically stabilized even for pressures above 56 GPa for the complex structural phase transformations. Specifically, we propose that the Li metal is likely to crystallize in the *I*4/*mcm* structure at high temperature.

Furthermore, the *I*4/*mcm* structure is predicted to be the thermodynamically stable structure at high pressure. As displayed in the inset in Fig. 2, the *I*4/*mcm* structure become more favorable than the fcc structure above 110 GPa. This is due to the fact that the enthalpy of fcc structure is lower than that of the *I*4/*mcm* structure at low pressure but higher at high pressure. Interestingly, our calculations indicate the pressure-induced phase transitions in the fcc structure can transform into the complex structures (*C*2*mb*, *C*2*cb*, and *I*4/*mcm*).

**Figure 3:**
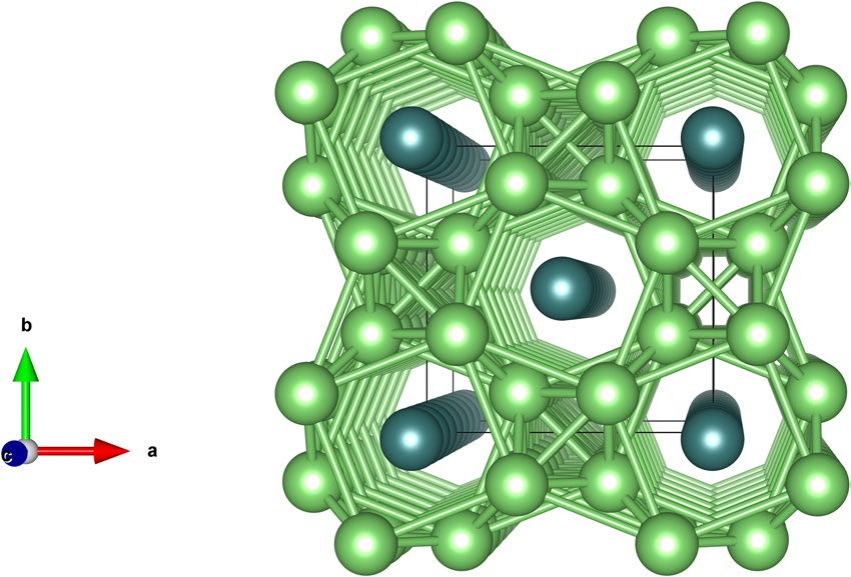
The crystal structure of the host-guest structure of Li. The host structure (dark green atoms) with guest chains (light green atoms) is shown in a c-axis projection.

Figure 3 displays a schematic of the HG structure at 150 GPa. We have described in greater detail, the HG structure is a commensurate supercell in along the *c* axis by approximating *c*_*H*_/*c*_*G*_ = 4/3 and component of the guest structure made up of chains that lie in channels in along the *c* axis of the host structure. The optimized lattice parameters for the host structure are *a* = 4.525 Å and *c* = 2.856 Å with Li atoms located at 8 *h* symmetry sites of space group *I*4/*mcm* at (0.166, 0.666, 0) and Li atoms are placed at (0, 0, 0) and (0.5, 0.5, 0) in the guest structure.

**Figure 4:**
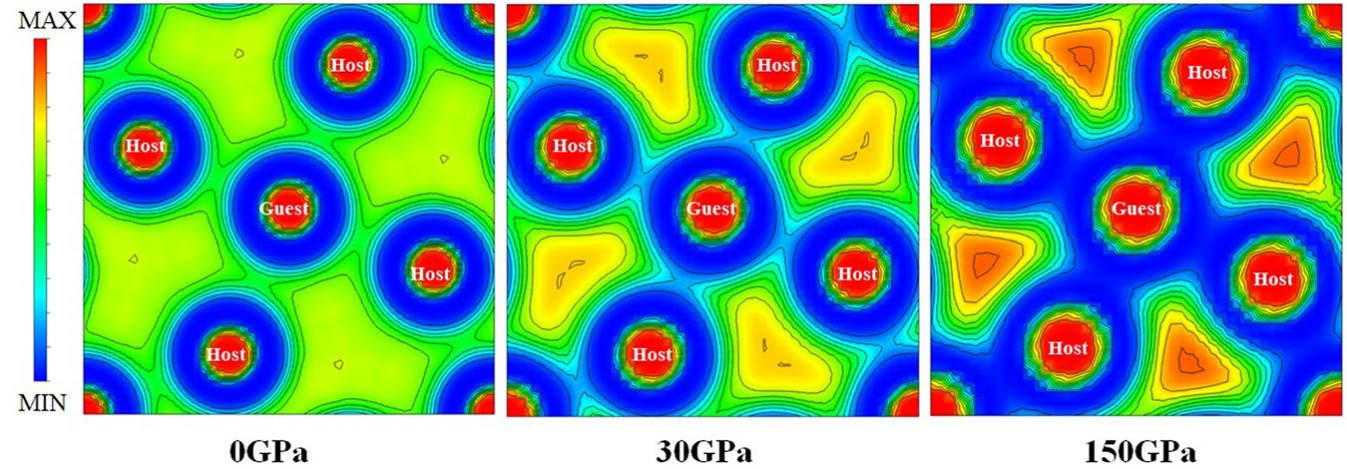
The electron localization function (ELF) in the (001) atomic plane of the host-guest structure of Li under pressures of 0, 30, and 150 GPa.

By analogy, *s* and *p*-state electrons of Li^20,21^ represent to the orbital quantum numbers which refer to *σ* and *π* states. However, the different characteristics of σ and *π* bond have presented in an electron localization function (ELF)^22,23^. The distances between the first (host-guest) and second (host-host) nearest neighbors (NN) are 2.927 Å and 4.140 Å at pressure 0 GPa, 2.252 Å and 3.185 Å at pressure 30 GPa, and 1.687 Å and 2.385 Å, at pressure 150 GPa, respectively. Displays a schematic of the HG structure combined with the ELF as can be seen Fig. 4). We have compared the ELF of the HG structure in the (001) atomic planes reveals the bonding network at pressures 0, 30, and 150 GPa, respectively. At ambient pressure, the electrons with the σ(*s*)-*π*(*p*) bonding is regularly distributed between the host-guest atoms and the host-host atoms. With increasing pressure, 30 GPa, the ELF display a slight decrease in distribution of electrons between the host-guest atoms and it displays the σ bond led to the weakly bonding^21^. While distribution of electrons is increased between the host-host atoms in this region, it is a strong localization electron of *π* bond. Obviously, the distribution of electrons becomes more rough at pressure 150 GPa. The accumulation of electrons between the host-host atoms shows that the bonds are the strongly formed in *π*(*p*) state. The bonding is favored in the *π* bond when the interatomic distance is shorter than 1.687 Å, which therefore contributes to the stability of the HG structure at high pressure^24^. According to the calculation of Neaton and Ashcroft^25^, they reported the localization of electron density interstitial regions with “pair” of electrons. The core region, which is the *s*-*pπ* hybridization state^25^, has led to the localization of electron density. Our calculation point out that the host-host atoms within the pair has a large core consisting of 1*s* electrons. With increasing pressure, the outer 2*s* electrons are pushed out of the *s*-*pπ* hybridization state. This remarkable result is unusual in the understanding that there is truly no the *s*-*pπ* hybridization state between the host-guest atoms but there is the *s*-*pπ* hybridization state between the host-host atoms. Also, it is useful to point out that it can be an incommensurate HG structure. In addition, our study gives the excellent account of the experimental observation^14^. The interpretation of the bonding nature is examined by analyzing the ELF. First, we observe that the pairing of electrons in the HG structure is a covalent bond^26^. Second, we see that the calculation of ELF shows the electron localization of *π* bonding between the host-host atoms, which would tend to favour superconductivity at higher presssure. Therefore, we strongly suggest that there are the strongly localized electrons between the host-host atoms, which is form *π* bonding, and it leads to superconducting phase. Hence, the question of whether metallic HG structure is a superconductor can only be answered by the experimental observation.

**Figure 5:**
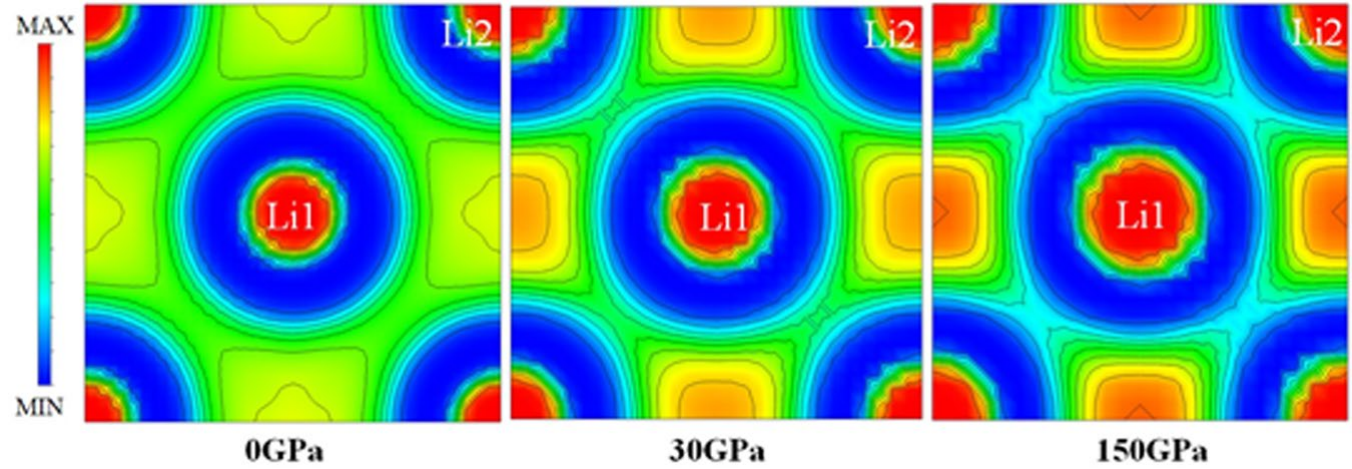
The electron localization function (ELF) in the (001) atomic plane of the fcc structure of Li under pressures of 0, 30, and 150 GPa.

Moreover, the transition mechanism from the fcc to HG structures is proposed by describing the crystallographic groups. It shows that the HG structure is the thermodynamically stable structure. At this point, we can examine the transition mechanism by the analysis of local changes of coordination in the (001) atomic plane. Notably, the ELF reveals the influences of pressure on the chemical bonding that it is given to clarify the electronic origins of the stability of the HG. This is because the electron localization tends to accumulate between the host-host bonds in Li framework. It is obvious that electrons tend to move away from the host-guest bonds (ambient pressure) to the host-host bonds (high pressure), which has been observed by the local changes of coordination in the (001) atomic plane. This is due to the fact that the accumulation of the electron localization increases according to the increasing pressure. Especially, the accumulation of the electrons could be delivered through the high-density between the host-host bonds at 150 GPa as can be seen in Fig. 4. Likewise, the the fcc structure, the analysis of local changes of coordination in the (001) atomic plane is compared at 0, 30, 150 GPa as shown in Fig. 5, the influences of pressure on the chemical bonding of the fcc structure shows that the electrons are regularly distributed between the Li1-Li1 bonds and Li1-Li2 bonds at 0 GPa. With increasing pressure, the distribution of electrons is slightly increased between the Li1-Li1 bonds and decreased between the Li1-Li1 bonds at 30 GPa. In addition, at 150 GPa, the distribution of electrons between Li1-Li1 bonds is more accumulated, while a slight decrease in the distribution of electrons between Li1-Li2 bonds is observed. Certainly, the electrons move away from the Li1-Li2 bonds (ambient pressure) to Li1-Li1 bonds (high pressure) as well as the Li1-Li1 bonds shows the appearance of the metallic bonds. In fact, the accumulation of the electron localization increases according to the increasing pressure, therefore the accumulations of electrons to the interstitial sites of the lattices of the HG and fcc structures may account for their transitions mechanism at high pressure. The interpretation of the bonding nature indicated that the covalent bonds in the HG structure can be characterized as a metallic bonding. Moreover, a remarkable property of the fcc structure is the metallic phase as well, thus giving rise to a metal-metal transition, which can be explained by the fcc-HG transformation.

**Figure 6:**
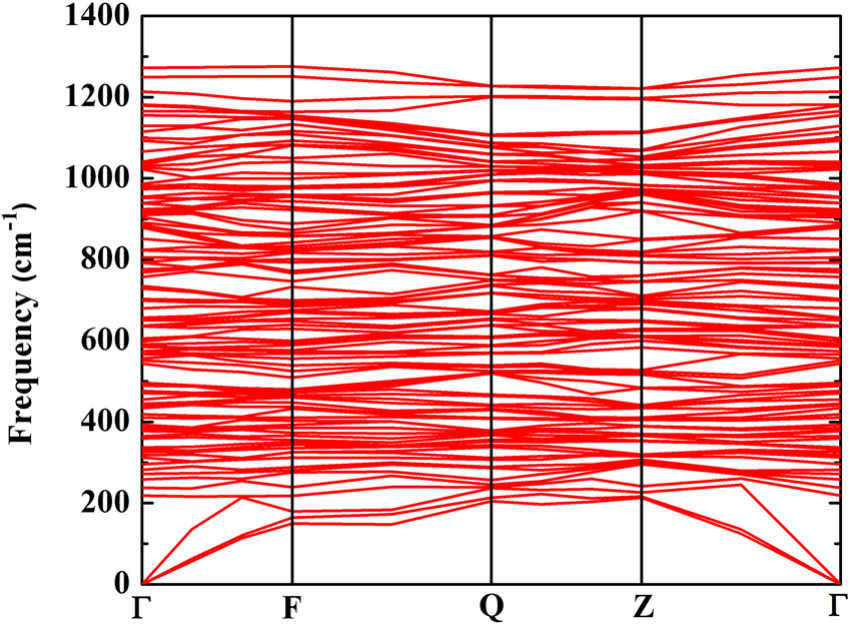
The dynamical harmonic stabilization of the HG structure at pressure 180 GPa.

To investigate the stability of Li, we have found that the commensurate value of 4/3 is the most stable above pressure 110 GPa (see in Fig. 2). Here we calculate the dynamical harmonic stabilization of the commensurate value of 4/3 of the HG structure. The dynamical stability as obtained from the phonon dispersion relation is analyzed as a function of pressure (Fig. 6). At pressure180 GPa, the lack of imaginary frequencies ensures a dynamically stability of Li metal in HG structure. We also observe high-frequency band from our calculation which we believe that it gives a significant contribution to the strongly localized electron of *π* bond. Moreover, the distances between the host-host atoms indicate the hardening phonon and it has also led to the stabilization of the HG structure.

## Conclusion

In summary, the AIRSS calculation is performed to predict a new stable the HG structure of Li above 110 GPa. We propose the complex structural phase transformations at finite temperature and high pressure in which the HG structure is the ground state structure and stable beyond the pressure of above 110 GPa. It should be noted that the fcc structure can generate the HG structure at a finite temperature and high pressure. The dynamical harmonic stabilization unveils the dynamically stable structure of Li metal at 180 GPa. The distribution of electrons between the host-host atoms is strongly localized electron of *π* bond which has led to the stability of the HG structure. This remarkable result confirms that Li can form the HG structure under high pressure and high temperature which put the HG structure to be a common high-pressure structure among alkali metals.

## Methods

We have used the AIRSS technique^19^ and *ab initio* calculation of the Cambridge Serial Total Energy Package (CASTEP)^27^ to predict the HG crystal structure of Li under high pressure. The AIRSS technique is the high potential for the prediction of the materials. For example, it has been used to predict the crystal structures in material science such as hydrogen^28,29^, helium^30^, lithium^17^, carbon^31^, nitrogen^32–34^, oxygen^35^, aluminium^18^, strontium^36^, metal hydrides^19,37–39^, and molecular crystals^40–42^.

We have performed the AIRSS calculation to aid purify the high-pressure phase of Li. Regarding the first type, we place Li atom randomly within the cells and relax cell by shaking. In the second type, we place atoms within the cells using preliminary experimental study^43^ with constraints on the crystal symmetry^29,31^. The cell shapes and atomic positions are then relaxed to the ground state structure at each pressure. We consider simulation cells containing 2, 4, 6, 8, 10, 11, 12, 14, 15 and 24 atoms of Li at pressures 20, 30, 40, 60, 80, 100, and 200 GPa. These findings help to space out the atoms suitably while retaining a high degree of randomness. A plane-wave basis-set energy cutoff of 270 eV and an initial Brillouin-zone (BZ) sampling grid of spacing 2*π* × 0.07 Å^−1^ were found to be sufficient for the initial search over structures^19,29,32,37^. The generalized gradient approximation (GGA) with the Perdew-Burke-Ernzerhof (PBE) parametrization^44^ for the exchange-correlation functional to density functional theory is used for the structure searches.

We have here presented the structures at low and high pressures using the GGA-PBE^44^ for the exchange-correlation functional to density functional theory. We employ the projector augmented wave (PAW) method^45^, as implemented in the Vienna *ab initio* simulation package (VASP)^46^. The PAW potential with a 3-electron (1*s*2*s*) for Li has been employed with plane waves basis set up to a cutoff energy of 600 eV and the initial BZ sampling grid of spacing 2*π* × 0.02 Å^−1^. The structures are relaxed using the conjugate gradient scheme. All considered structure are relaxed at each pressure until the Hellman-Feynman forces became less than 10^−3^ eV/Å.

The structures are relaxed and their equations of state are obtained by fitting with the calculated energy-volume data to the third-order Birch-Murnaghan equation. We then calculate the enthalpy-pressure relationship and the high-pressure phase of the experimental information^10^.

The existence of the HG structure presented here is based on the GGA-PBE using *ab initio* lattice dynamics with finite displacement method as well as supercell scheme by CASTEP^27^.

LaTeX formats citations and references automatically using the bibliography records in your. bib file, which you can edit via the project menu. Use the cite command for an inline citation, e.g.^1–46^.
